# Local therapy treatment conditions for oligometastatic non-small cell lung cancer

**DOI:** 10.3389/fonc.2022.1028132

**Published:** 2022-12-08

**Authors:** Suli Zhang, Qian Sun, Feng Cai, Hui Li, Yufu Zhou

**Affiliations:** ^1^ Department of Radiation Oncology, First Affiliated Hospital, Bengbu Medical College, Bengbu, Anhui, China; ^2^ Department of Nuclear Medicine, First Affiliated Hospital, Bengbu Medical College, Bengbu, Anhui, China

**Keywords:** non-small cell lung cance, oligometastasis, local ablation therapy, radiation therapy, stereotactic ablation body radiation therapy, treatment conditions

## Abstract

Standard treatments for patients with metastatic non-small cell lung cancer (NSCLC) include palliative chemotherapy and radiotherapy, but with limited survival rates. With the development of improved immunotherapy and targeted therapy, NSCLC prognoses have significantly improved. In recent years, the concept of oligometastatic disease has been developed, with randomized trial data showing survival benefits from local ablation therapy (LAT) in patients with oligometastatic NSCLC (OM-NSCLC). LAT includes surgery, stereotactic ablation body radiation therapy, or thermal ablation, and is becoming an important treatment component for OM-NSCLC. However, controversy remains on specific management strategies for the condition. In this review, we gathered current randomized trial data to analyze prognostic factors affecting patient survival, and explored ideal treatment conditions for patients with OM-NSCLC with respect to long-term survival.

## Introduction

Nearly half of non-small cell lung cancer (NSCLC) patients have metastases at diagnosis (1). In recent years, metastatic NSCLC treatment predominantly included palliative chemotherapy and radiotherapy, with 2-year survival rates of only 10% (2, 3). Recent advances in immune checkpoint inhibitor and targeted drug strategies have prolonged the survival of patients with NSCLC. In stage IV NSCLC, the 5-year survival rate with pembrolizumab is 32% in patients with high programmed death-ligand 1 (PD-L1) expression (4), while in those with anaplastic lymphoma kinase (ALK) rearrangement (5), the 5-year survival rate with alectinib is 62%. FLAURA trial data (6) showed that the median overall survival (OS) for osimertinib in advanced epidermal growth factor receptor (EGFR)-mutated NSCLC was 38.6 months. However, targeted therapies will develop resistance, with most patients eventually succumbing to progressive and fatal disease.

Based on Halsted’s cancer spread theory (7), in 1995, Hellman and Weichselbaum proposed the concept of oligometastasis, which is considered a transitional state between localized and extensive metastases (8). Retrospective studies reported that progression sites in advanced NSCLC patients were mainly manifested by the progression of existing metastatic sites (9, 10), which suggested that local metastases treatment may prolong patient progression-free survival (PFS) and further improve the quality of life, and even prolong patient survival times. Prospective single-arm (11–15) and retrospective studies (16, 17) successively showed that local ablation therapy (LAT) for oligometastatic NSCLC (OM-NSCLC) improved patient PFS and OS. Subsequent data from three Phase II randomized trials (18–22) and one phase III randomized controlled trial (23) showed that local therapies toward oligometastatic lesions benefited patient survival. Due to different enrollment criteria, treatment modalities, and low numbers of patients with OM-NSCLC in these trials, it is difficult to draw pooled conclusions. To design future studies and maintain consistency across trials, in 2020, the European Society for Radiotherapy and Oncology (ESTRO) and the European Organization for Research and Treatment of Cancer (EORTC) selected 17 characteristic oligometastatic disease (OMD) factors using the Delphi consensus process, and established a decision tree and nomology for OMD classification (24). The consensus defines the status of OMD, such as synchronous oligometastases vs. metachronous oligometastases, and oligopersistent disease vs. oligoprogressive disease. The general consensus defines synchronous oligometastases as metastases appearing within 6 months of a primary tumor diagnosis, while metachronous oligometastases appear 6 months after a primary tumor diagnosis. It is possible that the biological status of synchronous and metachronous oligometastases is different, with some studies showing that patients with synchronous oligometastases have lower survival rates (25). Based on the available data, in the same year, ESTRO and the American Society for Radiation Oncology (ASTRO) defined OMD as controlling the primary lesion, concomitant with the safe treatment of 1–5 metastatic lesions (26). At present, OM-NSCLC is in exploratory stages, and disease management remains challenging. Since diagnosis depends on imaging methods, we screen appropriate patient populations by identifying patients, tumors, and treatment characteristics, so as to optimize clinical decision-making and allow more patients to benefit from local treatments.

In this review, we used PubMed, from inception to September 1^st^ 2022, to identify randomized controlled trials, cohort studies, and review articles related to local treatment in OM-NSCLC patients. The literature search was limited to articles in English. The main search terms were: LAT/LCT (“ablative” or “ablation” or “consolidation” or “consolidative” or “stereotactic” or “SABR” or “SBRT”) in metastatic non-small cell lung cancer (“oligo-” or “metasta- “or” advanced”) and (“NSCLC”). We also queried ongoing randomized controlled trials using ClinicalTrials.gov.

Inclusion criteria: 1. Metastatic NSCLC was confirmed by histology or cytology, had a limited number of metastases (≤10 metastatic lesions). 2. Patients had measurable lesions at baseline which were suitable for local treatment. 3. All metastatic lesions were treated locally. 4. The findings included PFS or OS or adverse events (AEs).

Exclusion criteria: 1. >10 metastatic lesions. 2. A history of prior malignancy that potentially interfered with treatment options (no evidence of disease for at least 1 year). 3. The patient’s past or present participation in another study affects the results of this study.

## Randomized clinical trials investigating local ablation therapy for OM-NSCLC

Gomez et al. (18, 19) performed a randomized multicenter phase II clinical trial with the following inclusion criteria: (1) pathologically confirmed stage IV NSCLC and (2) patients with no progression and 1–3 metastases after first-line systemic chemotherapy - the Easten Cooperative Oncology Group, (ECOG) score was 0–2. The control group comprised patients receiving maintenance systemic therapy or undergoing observations, while the study group received local consolidation therapy (LCT) (radiation or surgery) with or without subsequent maintenance therapy. The data showed that PFS was significantly improved in the LCT group, with PFS of 11.9 months and 3.9 months in study and control groups, respectively, after a median follow-up of 12 months (*P*=0.0054). Long-term follow-up showed that the median OS was 41.2 months in the LCT group and 17.0 months in the control group (*P*=0.017). Research showed 20% grade 3 toxicity levels in the LCT group when compared with 8% the control group. No grade 4–5 toxicity was observed.

After this trial was closed, Iyengar et al. (20) performed a single-institution randomized phase II trial and enrolled 29 patients with similar criteria. Oligometastases were defined as 1–5 metastases after receiving induction systemic therapy, and patients were randomized to two groups; the control group received maintenance chemotherapy only, while the stereotactic ablative radiotherapy (SABR) group received SABR and maintenance chemotherapy. The PFS in SABR and control groups was 9.7 months and 3.5 months, respectively (*P*=0.01). Toxicity levels were similar between groups.

SABR-COMET (21, 22) is a multi-institution randomized phase 2 study, which required that the primary tumor had to be definitively treated 3 months before enrollment, with no disease progression by imaging and oligometastases defined as 1–5 metastatic lesions. Randomly assigned patients (1:2) received standard treatment (control group) or standard treatment + SABR (SABR group). The results showed that SABR was associated with improved OS, but adverse events of grade 2 or above in the SABR group were 20% more than in the control group, and three treatment-related deaths occurred. Long-term follow-up showed that the 5-year OS was 17.7% (95% confidence interval (CI): 6%–34%) in the control group vs. 42.3% (95% CI: 28%–56%, *P*=0.006) in the SABR group, with no new 2–5 grade adverse events.

The Phase III randomized controlled trial SINDAS(NCT 02893332) (23) included patients with synchronous OM-NSCLC with EGFR mutations, ≤ 5 metastatic lesions, with participants randomly assigned to receive first- generation tyrosine kinase inhibitors (TKIs) alone or upfront SABR plus TKIs for all disease sites. Interim analyses showed that the PFS in the TKIs alone group was 12.5 vs. 20.2 months in upfront SABR combined with TKIs group (*P*<0.001), OS was 17.4 vs. 25.5 months (*P*<0.001), and adverse events were similar between groups.

Based on these randomized controlled trials, LAT demonstrated significant clinical benefits in OM-NSCLC patients with EGFR mutations, and also wild-type OM-NSCLC patients. Failure patterns in control and experimental groups in the first three OM-NSCLC randomized controlled studies showed that local treatment blocked the progression of existing metastatic sites and prolonged PFS. Studies by Gomez et al. (18, 19) and Palma et al. (21, 22) showed that both initial and later salvage local treatments improved OS. In Gomez et al. (18, 19), the emergence time of new lesions in the local treatment group was longer when compared with the maintenance treatment group (*P*=0.0497), indicating that local treatment may have altered the natural disease course by limiting potential spread, or promoting the long-term control of subclinical lesions by altering antitumor immune responses. In Kwint et al. (27), five patients (5%) were still alive after 3 years of treatment, without any sign of recurrence. De Ruysscher et al. (12) observed that six patients (15.4%) had no disease progression after 2 years. These data support the concept of OMD in NSCLC and show that these patients may be cured or have good PFS outcomes. In combination with previous research data,we analyzed patient and tumor characteristics (including patient age, gender, ECOG score, pathological type, tumor size, lymph node metastasis, gene mutation status, number and location of metastases and oligometastatic status: synchronous oligometastases vs. metachronous oligometastases), and also investigated intervention measures (including the timing of LAT treatment, whether local therapy combined with systemic therapy, type of systemic therapy, dose regimens of radiotherapy, etc.).

## LAT for OM-NSCLC patients generates ideal conditions for long-term survival

### Effective systemic therapy and the radical treatment of primary tumors are crucial for patients with oligometastases

Previous studies reported that most patients with oligometastases, as detected by imaging, had undetected micrometastases, and as ablation therapy could only treat visible lesions, effective systemic therapy was essential to control micrometastases. This was demonstrated by Gomez et al. (18, 19) and Iyengar et al. (20); patients received systemic therapy prior to enrollment, but were not enrolled after disease progression. In these studies, both patient groups experienced distant progression after treatment, suggesting that micrometastases were present in patients before oligometastases were visualized using local therapy. This was also shown by monitoring micrometastases in the SABR-COMET study (21, 22). Furthermore, as observed by Gomez and Iyengar, patients with early disease progression on systemic therapy were less likely to gain much benefit from LAT when compared with patients with systemic therapy-responsive disease. Interim analysis results from a Phase III randomized controlled trial by Wang et al. (28) showed that first-generation TKIs combined with SABR benefited patient survival. The ongoing NORTHSTAR study (NCT 03410043) is currently investigating ositinib efficacy with or without local treatment in patients with stage IV EGFR-mutated NSCLC.

None of these randomized trials provided evidence of immunotherapy combined with local therapy. Studies have shown that approximately 20% of NSCLC patients benefit from immunotherapy (29, 30), and radiotherapy can ameliorate the tumor microenvironment to enhance immunotherapy efficacy, thereby achieving a synergistic effect. In 2021, Willemijn et al. (31) pooled and analyzed PEMBRO-RT and MDACC trials, and showed that pembrolizumab combined with radiotherapy had beneficial effects on patient survival. The median PFS in the pembrolizumab group was 4.4 months, while pembrolizumab combined with radiotherapy was 9.0 months (*P*=0.045); the median OS in the pembrolizumab group was 8.7 months, but 19.2 months in the pembrolizumab plus radiotherapy group (*P*=0.0004), with no new safety concerns.

In the SABR-COMET study, authors required that primary tumors in patients were radically treated 3 months before enrollment, and that patients could be enrolled after no disease progression by imaging. In 2018, Petrelli et al. (32) conducted a meta-analysis of radiotherapy for primary tumors in synchronous OM-NSCLC and showed that 1-, 3-, and 5-year survival rates in patients who received primary tumor radiotherapy were 70%, 29%, and 20%, respectively. This therapy improved PFS (*P*<0.001) and OS (*P*<0.001) in patients with synchronous OM-NSCLC. Preclinical data showed that primary radical radiation therapy enhanced the presentation of tumor-associated antigens.

SABR can be performed at multiple tumor sites with low toxicity, and does not delay systemic therapy. In the systematic review and meta-analysis by Lehrer et al. (33), SABR generated satisfactory results in local control (LC), OS, and PFS at 1 year after treatment, and the incidence of early and late grade 3–5 toxic events was usually < 10%. Therefore, SABR has become a primary treatment modality for unresectable OM-NSCLC (34). SABR dose regimens are usually based on lesion location and size, and also their proximity to adjacent organs at risk. Treatments generally range from 1–5 fractions, but depending on proximity to the critical structure, hypofractionated radiotherapy from 5-15 fractions may be considered. The multidisciplinary American Radium Society Lung Cancer Panel (35) proposed that primary lung tumors or metastases (< 5 cm) outside the “central area” could be administered 34 Gy/fraction, 45–54Gy/3 fractions, 48–50Gy/4 fractions, and 50–60Gy/5 fractions. Central regions include tumors within approximately 2 cm of the proximal bronchial tree and adjacent to mediastinal or pericardium, heart, brachial plexus nerve, esophagus, spinal cord, major vessels, recurrent laryngeal nerve and phrenic nerve. A central primary tumor or larger metastasis (> 5 cm) can be treated with the following regimens: 50 Gy/4–5 fractions, 60 Gy/8 fractions, and 70 Gy/10 fractions. Hypofractionated radiotherapy (8–15 fractions) is usually recommended for ultracentral tumors. For parallel organs, such as the liver and adrenal glands, if the target range does not include structures such as the gastrointestinal tract (GI), treatment options using aforementioned lung protocols may be considered. Otherwise, the dose should be adjusted to keep GI within tolerance limits. For bone metastases, 12–16 Gy/fraction for non-spinal lesions, 12–24 Gy/fraction for spinal lesions, or 24 Gy/2 fractions are recommended. For patients with brain metastases, according to current consensus guidelines (36, 37), it is recommended to use stereotactic radiosurgery (SRS) or hypofractionated stereotactic radiotherapy (HSRT) alone for patients who cannot undergo surgery, have 5–10 brain metastases, and a cumulative tumor volume of < 7 mL. Gutschenritter et al. (38) also suggested that for large brain metastases or resected cavities, 3- or 5-fraction schemes should be used instead of SRS to provide 15 Gy or smaller SRS schemes so as to reduce the risk of radiation necrosis, and improve local control rates. For patients with OMD who can be treated surgically, postoperative SRS is currently considered the standard treatment for all surgically resected brain metastases (39, 40). However, postoperative SRS also has some disadvantages, such as delayed radiotherapy for postoperative complications, expansion of the surgical tumor bed to obtain the best local control rates, dynamic changes in the resection cavity between postoperative magnetic resonance imaging (MRI) and SRS treatment plan MRI, etc. As a new treatment mode, preoperative SRS avoids these limitations and reduces the risk of leptomeningeal disease while maintaining LC (41, 42). However, as a new treatment method, the approach requires much more research to fully characterize its advantages and disadvantages.

### Tumor burden may be a major factor affecting prognosis

Potential tumor burden traits can include the number and location of metastases, and the location of lymph nodes. Although no current standard definitions of optimal tumor burden exist, the literature generally indicates 1–5 metastases as a reasonable number for LAT. Therefore, accurate staging before treatment to reduce missing lesions is crucial. Positron emission computed tomography (PET)/computer tomography (CT) showed high sensitivity for staging mediastinal lymph nodes and detecting distant and occult metastases. When combined with brain MRI, oligometastatic disease detection was more accurate (43). In their oncogene-mutant NSCLC study, Ng et al. (44) used PET/CT to show higher sensitivity in detecting oligoprogressive disease (81.3% vs. 68.6%). Two trials (45, 46)required PET/CT and a contrast-enhanced MRI of the brain as baseline scores, which are recommended by the EORTC consensus (47).

In 2016, Ashworth et al. (25) conducted a LAT systematic review and meta-analysis in 757 patients with OM-NSCLC from 20 centers; they analyzed risk factors and performed risk stratifications, and showed the low-risk group included no lymph node metastasis (N0) and metachronous oligometastases, while the 5-year OS was 47.8%. The intermediate-risk group with N0 with synchronous oligometastases had a 5-year OS of 36.2%. Finally, the high-risk group with N1 or N2 with synchronous oligometastases had a 5-year OS of 13.8%.

Brain metastases are challenging in terms of selecting patients for local oligometastases treatments. Data has shown that approximately 10% of patients with advanced NSCLC have brain metastases at diagnosis, while 40% will progress to brain metastases (48). For patients with EGFR mutations and ALK rearrangements, this number is as high as 60% (49, 50). Standard cytotoxic drugs cannot easily penetrate the blood-brain barrier; patients with NSCLC and brain metastases have a poor prognosis with a median OS of approximately 7 months (51).

### LAT timing

There is no clear consensus on the timing for LAT, nor is it clear if intracranial and extracranial metastases are similar or distinct disease states, but previous studies can shed light on this. Trials by Gomez and Iyengar showed significantly longer median PFS and OS rates in LCT groups when compared with control groups. Notably, LCT was performed after induction therapy (18, 21). Additionally, a retrospective study (52) of EGFR-mutant NSCLC patients with brain metastases showed that when compared with previous whole-brain radiotherapy (WBRT) or EGFR-TKIs, SRS after treatment with TKIs achieved longer survival, and the median OS in SRS, WBRT, and EGFR-TKIs cohorts were 46, 30, and 25 months, respectively (*P*=0.001).

While there is progress after systemic therapy, upfront LAT prevents further single and multiple metastases at the initial oligometastatic site. In a Phase III randomized controlled trial by Wang et al. (28), both PFS and OS benefited by upfront SABR combined with the TKIs group, with similar adverse events identified between both groups. A prospective one-arm phase II study by Bauml et al. (53) enrolled patients with synchronous oligometastases (metastases present at initial diagnosis) and metachronous oligometastases (oligometastases which gradually developed after local primary lesion treatment) to receive pembrolizumab for 8–16 cycles within 4–12 weeks after LAT treatment. Their results showed a median PFS of 19.1 months vs. 6.6 months (historical; *P* = 0.005). The 1 year OS rate was 90.9% and the 2 year OS rate was 77.5%. Pembrolizumab after LAT did not generate new safety issues or reduce the patients’ quality of life. However, as mentioned (18–22), LCT results after systemic therapy were equally reassuring. A large, ongoing Phase III randomized controlled trial, OITROLC (NCT 02076477), is currently enrolling 1–5 patients with synchronous oligometastases; patients in the upfront LAT group are receiving concurrent chemoradiotherapy followed by chemotherapy for two cycles, and patients in the other group are receiving neoadjuvant chemotherapy every 3-4 weeks for two cycles followed by concurrent chemoradiotherapy. Critically, data from this study will help clinicians understand the optimal timing of local and systemic treatments.

Data from a mouse study by Dovedi et al. (54) showed that PD-L1 expression increased within 24 hours after radiotherapy cycle completion, and remained elevated for at least 7 days afterwards. To reduce delays in systemic therapy, Theelen et al. (55) used pembrolizumab maintenance therapy within 1 week after radiotherapy completion. We hypothesize that LCT before systemic therapy or early LCT after systemic therapy has a better prognosis, but further research is required to confirm this.

### Patient life expectancy, health status, gene mutation status, and internal medicine are important factors

Clinical trials often require patients to have a life expectancy of ≥ 6 months, e.g., SABR-COMET. Additionally, physical status scores, such as ECOG or Karnofsky performance status (KPS) scores are used to indicate a patient’s health status. Jang et al. (56) analyzed 1655 patients with advanced cancer and found that an ECOG score = 0 was associated with a median OS of 293 days, while 1 was associated with a median OS of 197 days. The median OS for ECOG 2–4 patients was 104, 55, and 25.5 days, respectively. The median OS scores for KPS were: KPS 80–100 = 215 days; KPS 60–70 = 119 days; KPS 40–50 = 49 days; and KPS 10–30 = 29 days. Therefore, patients with ECOG scores of 2–4 or KPS ≤60 had poor prognoses for local therapy, as were patients with comorbid medical disease. Sperduto et al. (57) updated the lung-molGPA prognostic index, which is a scoring system for patients with NSCLC and brain metastases, and is based on a previous diagnosis-specific graded prognostic assessment, except for patient age, KPS, extracranial metastases, and brain metastases. In addition to the number, EGFR and ALK mutational status in adenocarcinoma patients was added. The overall median survival of the study cohort was 12 months, and the median survival of patients with a lung-molGPA score of 3.5–4.0 was nearly 4 years.

## LAT safety in oligometastatic NSCLC

Radiation therapy has been used in studies investigating the role of LAT in oligometastases. Dosing/fractionation varied, but overall, the results supported an adequate safety profile for treating multiple disease sites. In the original SABR report, Salama et al. (58) treated 29 patients with 56 metastases from November 2004 to March 2007. Two patients experienced grade 3 or higher toxicity levels, including one patient with radiation pneumonitis and one with persistent nausea/vomiting. Of these examples, lung injury may be a potentially fatal event. A recent prospective clinical trial (59) showed that patients treated with EGFR-TKIs + stereotactic body radiation therapy (SBRT) were more likely to develop radiation pneumonitis (25% vs. 0%) when compared with the SBRT alone treatment group, with 4/20 patients developing grade 2 and above pneumonia. However, due to the small number of patients and no observed statistical differences, it was difficult to conclude that EGFR-TKIs combined with Radiation Therapy (RT) increased the risk of radiation-induced lung injury. Future research should focus on the stratification of these factors to formulate more personalized treatment plans.

## Ongoing randomized trials

Randomized trial evidence for local therapy in OM-NSCLC is rapidly expanding, with multiple ongoing randomized controlled trials (**Table 1**). NRG-LU002 (NCT 03137771) and SARON (NCT 02417662) are investigating synchronous OM-NSCLC. NRG-LU002 and SABR-COMET-3 (NCT 03862911) enrolled patients with 1–3 oligometastases, SARON included patients with 1–5, and SABR-COMET-10 (NCT 03721341) included 4–10. The STOP study (NCT 02756973) is a multicenter randomized phase II trial of 90 patients with 1–5 oligoprogressive NSCLC. The HALT study (NCT 03256981) included 110 patients who progressed after TKI therapy. TRAILOCLORI01 (NCT 05111197) was designed to evaluate the effects of radiation therapy on OS in patients with OM-NSCLC who were treated with first-line immunotherapy (or combined with chemotherapy). The NIRVANA-LUNG (NCT 03774732) randomized phase III trial evaluated the efficacy of LCT in combination with immunotherapy and chemotherapy in patients with locally advanced or metastatic NSCLC.

**Table 1:** Ongoing randomized trials.

Enrolling Study	Phase	Conditions	Synchronous or Metachronous	Oligometastatic Defifinition	Est. Enrollment	Driver mutations	Brain metastases	FDG-PET CT	Eligibility Criteria for Randomisation	Control Arm	Experimental Arm	Primary Outcomes
SABR-COMET-3 (NCT03862911)	III	multiple types of cancer	NR	1-3 metastases (all histologies)	297	NR	YES	Not mandatory	Controlled primary tumor	SOC	SABR + SOC	OS
SABR-COMET-10 (NCT03721341)	III	multiple types of cancer	NR	4-10 metastases (all histologies)	159	NR	YES	Not mandatory	Controlled primary tumor	SOC	SABR + SOC	OS
NRG-LU002 (NCT03137771)	II or III	NSCLC	NR	1-3 metastases	400	included	YES	Not mandatory	No PD after induction systemic therapy	SOC	SBRT+SOC	PFS (phase II) OS (phase III)
SARON (NCT02417662)	III	NSCLC	Synchronous	1-3 metastases	340	NO	YES	before inclusion	No PD after 2 cycles of systemic therapy	SOC	SOC Conventional RT +SABR	OS
HALT trial (NCT03256981)	II or III	NSCLC	NR	1-3 oligo-progressive lesions	110	ALL	NO	Not mandatory	NR	TKI	SBRT+TKI	PFS
TRAILOCLORI01 (NCT 05111197)	III	NSCLC	NR	1-5 metastases	112	NR	YES	before inclusion	No PD after immunotherapy	Immunotherapy	Immunotherapy + SRT	OS
STOP(NCT 02756793)	II	NSCLC	NR	1-5 oligo-progressive lesions	90	NR	YES	Not mandatory	No PD before the development of oligo-progressive lesions	SOC	SABR	PFS

NSCLC, non–small-cell lung cancer; G, toxicity grade; OS, overall survival; PFS, progression-free survival; SOC, standard of care; SRT, Stereotactic Radiation Therapy; TKIs, tyrosine kinase inhibitors; PD, progressive disease; NR, not reported.

## Other topical treatments

Before the advent of SRS and SBRT, surgical resection was the main treatment option for OMD. However, according to recent guidelines (60), there is limited evidence for surgery in OMD. Surgical intervention complications and the extent of resection required to complete an R0 resection require consideration. Although lung resection is associated with lower postoperative complications and mortality, it undoubtedly leads to higher complications and poorer quality of life than lobectomy or limited resection (61, 62).

Thanks to the introduction of minimally invasive techniques, several attempts have been made to use thermal ablation as a LAT for treating oligoprogressive and oligometastatic lesions in NSCLC. Thermal ablation therapy, including radiofrequency and microwave ablation, was initially used to treat various solid, liver, and lung tumor types (63, 64). Although no randomized evidence is available for OM-NSCLC, thermal ablation was successfully used to treat oligometastatic lesions in the liver, and an ongoing phase III COLLISION study (NCT 03088150) is comparing thermal ablation with surgical resection in colorectal cancer liver metastases (≤3cm) in terms of treatment efficacy. As with SABR, lesion size and location are critical parameters in choosing the best treatment modality. Therefore, treatment decisions must be individualized and made in multidisciplinary settings.

## Challenges and opportunities

Several unanswered questions persist in this controversial area. Firstly, metastasis numbers remain controversial. In prospective studies, the number of metastases was defined as 1–6, but the majority of selected patients had ≤ 2 metastatic disease lesions. Secondly, it is unclear if the primary tumor should be included and how to define mediastinal lymph nodes. Ongoing clinical trials are testing potential biomarkers, including circulating tumor DNA and circulating tumor cells, and also new imaging methods to better predict which patients truly have oligometastatic disease and will most likely benefit from ablation therapy. Finally, the optimal combination of ablation therapy with systemic therapy, including targeted therapy and immunotherapy, is an active, ongoing research direction.

## Conclusions

In conclusion, PFS and OS benefit have been shown after ablation therapy for OM-NSCLC. In the future, phase III data could provide better data to establish ideal treatment conditions for the disease. Also, in multidisciplinary environments, clinicians should continue to make their best clinical judgment and provide this kind of treatment in appropriate patients, usually those patients whose primary tumor is treated radically, who respond well to systemic treatment, have a low metastatic burden with metachronous oligometastasis, negative intrathoracic lymph nodes and good performance scores, and so on. Furthermore, the patient’s economic conditions, local medical access, and other factors must be considered throughout their medical journey.

## Author contributions

YZ and QS contributed to the scheme design and investigation. FC, HL and SZ contributed to the writing of the paper and submitted the final version. All authors contributed to the article and approved the submitted version.

## References

[1] Walters S Maringe C Coleman MP Peake MD Butler J Young N . Lung cancer survival and stage at diagnosis in Australia, Canada, Denmark, Norway, Sweden and the uk: A population-based study, 2004-2007. Thorax (2013) 68(6):551–64. doi: 10.1136/thoraxjnl-2012-202297 23399908

[2] Non-Small Cell Lung Cancer Collaborative G . Chemotherapy and supportive care versus supportive care alone for advanced non-small cell lung cancer. Cochrane Database Syst Rev (2010) (5):CD007309. doi: 10.1002/14651858.CD007309.pub2 PMC11380090 20464750

[3] Stevens R Macbeth F Toy E Coles B Lester JF . Palliative radiotherapy regimens for patients with thoracic symptoms from non-small cell lung cancer. Cochrane Database Syst Rev (2015) 1:CD002143. doi: 10.1002/14651858.CD002143.pub4 25586198 PMC7017846

[4] Brahmer JR Rodriguez-Abreu D Robinson AG Hui R Csőszi T Fülöp A . Lba51 keynote-024 5-year os update: First-line (1l) pembrolizumab (Pembro) vs platinum-based chemotherapy (Chemo) in patients (Pts) with metastatic nsclc and pd-L1 tumour proportion score (Tps) ≥50%. Ann Oncol (2020) 31:S1181–S2. doi: 10.1016/j.annonc.2020.08.2284

[5] Mok T Camidge DR Gadgeel SM Rosell R Dziadziuszko R Kim DW . Updated overall survival and final progression-free survival data for patients with treatment-naive advanced alk-positive non-Small-Cell lung cancer in the Alex study. Ann Oncol (2020) 31(8):1056–64. doi: 10.1016/j.annonc.2020.04.478 32418886

[6] Ramalingam SS Vansteenkiste J Planchard D Cho BC Gray JE Ohe Y . Overall survival with osimertinib in untreated, egfr-mutated advanced nsclc. N Engl J Med (2020) 382(1):41–50. doi: 10.1056/NEJMoa1913662 31751012

[7] Halsted WSI . The results of operations for the cure of cancer of the breast performed at the johns Hopkins hospital from June, 1889, to January, 1894. Ann Surg (1894) 20(5):497–555. doi: 10.1097/00000658-189407000-00075 PMC1493925 17860107

[8] Hellman S Weichselbaum RR . Oligometastases. J Clin Oncol (1995) 13(1):8–10. doi: 10.1200/JCO.1995.13.1.8 7799047

[9] Mehta N Mauer AM Hellman S Haraf DJ Cohen EE Vokes EE . Analysis of further disease progression in metastatic non-small cell lung cancer: Implications for locoregional treatment. Int J Oncol (2004) 25(6):1677–83. doi: 10.3892/ijo.25.6.1677 15547705

[10] Rusthoven KE Hammerman SF Kavanagh BD Birtwhistle MJ Stares M Camidge DR . Is there a role for consolidative stereotactic body radiation therapy following first-line systemic therapy for metastatic lung cancer? a patterns-of-Failure analysis. Acta Oncol (2009) 48(4):578–83. doi: 10.1080/02841860802662722 19373699

[11] Collen C Christian N Schallier D Meysman M Duchateau M Storme G . Phase ii study of stereotactic body radiotherapy to primary tumor and metastatic locations in oligometastatic nonsmall-cell lung cancer patients. Ann Oncol (2014) 25(10):1954–9. doi: 10.1093/annonc/mdu370 25114022

[12] De Ruysscher D Wanders R van Baardwijk A Dingemans AM Reymen B Houben R . Radical treatment of non-Small-Cell lung cancer patients with synchronous oligometastases: Long-term results of a prospective phase ii trial (Nct01282450). J Thorac Oncol (2012) 7(10):1547–55. doi: 10.1097/JTO.0b013e318262caf6 22982655

[13] Iyengar P Kavanagh BD Wardak Z Smith I Ahn C Gerber DE . Phase ii trial of stereotactic body radiation therapy combined with erlotinib for patients with limited but progressive metastatic non-Small-Cell lung cancer. J Clin Oncol (2014) 32(34):3824–30. doi: 10.1200/JCO.2014.56.7412 25349291

[14] Petty WJ Urbanic JJ Ahmed T Hughes R Levine B Rusthoven K . Long-term outcomes of a phase 2 trial of chemotherapy with consolidative radiation therapy for oligometastatic non-small cell lung cancer. Int J Radiat Oncol Biol Phys (2018) 102(3):527–35. doi: 10.1016/j.ijrobp.2018.06.400 PMC6743975 30003996

[15] Rusthoven KE Kavanagh BD Burri SH Chen C Cardenes H Chidel MA . Multi-institutional phase I/Ii trial of stereotactic body radiation therapy for lung metastases. J Clin Oncol (2009) 27(10):1579–84. doi: 10.1200/JCO.2008.19.6386 19255320

[16] Griffioen GH Toguri D Dahele M Warner A de Haan PF Rodrigues GB . Radical treatment of synchronous oligometastatic non-small cell lung carcinoma (Nsclc): Patient outcomes and prognostic factors. Lung Cancer (2013) 82(1):95–102. doi: 10.1016/j.lungcan.2013.07.023 23973202

[17] Hu F Xu J Zhang B Li C Nie W Gu P . Efficacy of local consolidative therapy for oligometastatic lung adenocarcinoma patients harboring epidermal growth factor receptor mutations. Clin Lung Cancer (2019) 20(1):e81–90. doi: 10.1016/j.cllc.2018.09.010 30341018

[18] Gomez DR Blumenschein GR Jr. Lee JJ Hernandez M Ye R Camidge DR . Local consolidative therapy versus maintenance therapy or observation for patients with oligometastatic non-Small-Cell lung cancer without progression after first-line systemic therapy: A multicentre, randomised, controlled, phase 2 study. Lancet Oncol (2016) 17(12):1672–82. doi: 10.1016/S1470-2045(16)30532-0 PMC5143183 27789196

[19] Gomez DR Tang C Zhang J Blumenschein GR Jr. Hernandez M Lee JJ . Local consolidative therapy vs. maintenance therapy or observation for patients with oligometastatic non-Small-Cell lung cancer: Long-term results of a multi-institutional, phase ii, randomized study. J Clin Oncol (2019) 37(18):1558–65. doi: 10.1200/JCO.19.00201 PMC6599408 31067138

[20] Iyengar P Wardak Z Gerber DE Tumati V Ahn C Hughes RS . Consolidative radiotherapy for limited metastatic non-Small-Cell lung cancer: A phase 2 randomized clinical trial. JAMA Oncol (2018) 4(1):e173501. doi: 10.1001/jamaoncol.2017.3501 28973074 PMC5833648

[21] Palma DA Olson R Harrow S Gaede S Louie AV Haasbeek C . Stereotactic ablative radiotherapy versus standard of care palliative treatment in patients with oligometastatic cancers (Sabr-comet): A randomised, phase 2, open-label trial. Lancet (2019) 393(10185):2051–8. doi: 10.1016/S0140-6736(18)32487-5 30982687

[22] Palma DA Olson R Harrow S Gaede S Louie AV Haasbeek C . Stereotactic ablative radiotherapy for the comprehensive treatment of oligometastatic cancers: Long-term results of the sabr-comet phase ii randomized trial. J Clin Oncol (2020) 38(25):2830–8. doi: 10.1200/JCO.20.00818 PMC7460150 32484754

[23] Wang X Bai YF Zeng M . First-line tyrosine kinase inhibitor with or without aggressive upfront local radiation therapy in patients with egfrm oligometastatic non-Small-Cell lung cancer: Interim results of a randomized phase iii, open-label clinical trial (Sindas) (Nct02893332). Int J Radiat Oncol Biol Phys (2020) 108(3):e81. doi: 10.1016/j.ijrobp.2020.07.1169

[24] Guckenberger M Lievens Y Bouma AB Collette L Dekker A deSouza NM . Characterisation and classification of oligometastatic disease: A European society for radiotherapy and oncology and European organisation for research and treatment of cancer consensus recommendation. Lancet Oncol (2020) 21(1):e18–28. doi: 10.1016/S1470-2045(19)30718-1 31908301

[25] Ashworth AB Senan S Palma DA Riquet M Ahn YC Ricardi U . An individual patient data metaanalysis of outcomes and prognostic factors after treatment of oligometastatic non-Small-Cell lung cancer. Clin Lung Cancer (2014) 15(5):346–55. doi: 10.1016/j.cllc.2014.04.003 24894943

[26] Lievens Y Guckenberger M Gomez D Hoyer M Iyengar P Kindts I . Defining oligometastatic disease from a radiation oncology perspective: An estro-astro consensus document. Radiother Oncol (2020) 148:157–66. doi: 10.1016/j.radonc.2020.04.003 32388150

[27] Kwint M Walraven I Burgers S Hartemink K Klomp H Knegjens J . Outcome of radical local treatment of non-small cell lung cancer patients with synchronous oligometastases. Lung Cancer (2017) 112:134–9. doi: 10.1016/j.lungcan.2017.08.006 29191586

[28] Wang XS Bai YF Verma V Yu RL Tian W Ao R . Randomized trial of first-line tyrosine kinase inhibitor with or without radiotherapy for synchronous oligometastatic egfr-mutated nsclc. J Natl Cancer Inst (2022):djac015. doi: 10.1093/jnci/djac015 PMC10248839 35094066

[29] Garon EB Rizvi NA Hui R Leighl N Balmanoukian AS Eder JP . Pembrolizumab for the treatment of non-Small-Cell lung cancer. N Engl J Med (2015) 372(21):2018–28. doi: 10.1056/NEJMoa1501824 25891174

[30] Brahmer J Reckamp KL Baas P Crino L Eberhardt WE Poddubskaya E . Nivolumab versus docetaxel in advanced squamous-cell non-Small-Cell lung cancer. N Engl J Med (2015) 373(2):123–35. doi: 10.1056/NEJMoa1504627 PMC4681400 26028407

[31] Theelen W Chen D Verma V Hobbs BP Peulen HMU Aerts J . Pembrolizumab with or without radiotherapy for metastatic non-Small-Cell lung cancer: A pooled analysis of two randomised trials. Lancet Respir Med (2021) 9(5):467–75. doi: 10.1016/S2213-2600(20)30391-X 33096027

[32] Petrelli F Ghidini A Cabiddu M Tomasello G De Stefani A Bruschieri L . Addition of radiotherapy to the primary tumour in oligometastatic nsclc: A systematic review and meta-analysis. Lung Cancer (2018) 126:194–200. doi: 10.1016/j.lungcan.2018.11.017 30527187

[33] Lehrer EJ Singh R Wang M Chinchilli VM Trifiletti DM Ost P . Safety and survival rates associated with ablative stereotactic radiotherapy for patients with oligometastatic cancer: A systematic review and meta-analysis. JAMA Oncol (2021) 7(1):92–106. doi: 10.1001/jamaoncol.2020.6146 33237270 PMC7689573

[34] Suh YG Cho J . Local ablative radiotherapy for oligometastatic non-small cell lung cancer. Radiat Oncol J (2019) 37(3):149–55. doi: 10.3857/roj.2019.00514 PMC6790793 31591862

[35] Amini A Verma V Simone CB 2nd Chetty IJ Chun SG Donington J . American Radium society appropriate use criteria for radiation therapy in oligometastatic or oligoprogressive non-small cell lung cancer. Int J Radiat Oncol Biol Phys (2022) 112(2):361–75. doi: 10.1016/j.ijrobp.2021.09.022 34571054

[36] Babu MA . Commentary: Congress of neurological surgeons systematic review and evidence-based guidelines on the use of stereotactic radiosurgery in the treatment of adults with metastatic brain tumors. Neurosurgery (2019) 84(3):E173–E4. doi: 10.1093/neuros/nyy602 30629268

[37] Milano MT Chiang VLS Soltys SG Wang TJC Lo SS Brackett A . Executive summary from American radium society’s appropriate use criteria on neurocognition after stereotactic radiosurgery for multiple brain metastases. Neuro Oncol (2020) 22(12):1728–41. doi: 10.1093/neuonc/noaa192 PMC7746939 32780818

[38] Gutschenritter T Venur VA Combs SE Vellayappan B Patel AP Foote M . The judicious use of stereotactic radiosurgery and hypofractionated stereotactic radiotherapy in the management of Large brain metastases. Cancers (Basel) (2020) 13(1):70. doi: 10.3390/cancers13010070 PMC7795798 33383817

[39] Brown PD Ballman KV Cerhan JH Anderson SK Carrero XW Whitton AC . Postoperative stereotactic radiosurgery compared with whole brain radiotherapy for resected metastatic brain disease (Ncctg N107c/Cec.3): A multicentre, randomised, controlled, phase 3 trial. Lancet Oncol (2017) 18(8):1049–60. doi: 10.1016/S1470-2045(17)30441-2 PMC5568757 28687377

[40] Mahajan A Ahmed S McAleer MF Weinberg JS Li J Brown P . Post-operative stereotactic radiosurgery versus observation for completely resected brain metastases: A single-centre, randomised, controlled, phase 3 trial. Lancet Oncol (2017) 18(8):1040–8. doi: 10.1016/S1470-2045(17)30414-X PMC5560102 28687375

[41] Patel KR Burri SH Asher AL Crocker IR Fraser RW Zhang C . Comparing preoperative with postoperative stereotactic radiosurgery for resectable brain metastases: A multi-institutional analysis. Neurosurgery (2016) 79(2):279–85. doi: 10.1227/NEU.0000000000001096 26528673

[42] Patel KR Burri SH Boselli D Symanowski JT Asher AL Sumrall A . Comparing pre-operative stereotactic radiosurgery (Srs) to post-operative whole brain radiation therapy (Wbrt) for resectable brain metastases: A multi-institutional analysis. J Neurooncol (2017) 131(3):611–8. doi: 10.1007/s11060-016-2334-3 28000105

[43] Lardinois D Weder W Hany TF Kamel EM Korom S Seifert B . Staging of non-Small-Cell lung cancer with integrated positron-emission tomography and computed tomography. N Engl J Med (2003) 348(25):2500–7. doi: 10.1056/NEJMoa022136 12815135

[44] Ng TL Morgan RL Patil T Baron AE Smith DE Ross Camidge D . Detection of oligoprogressive disease in oncogene-addicted non-small cell lung cancer using Pet/Ct versus ct in patients receiving a tyrosine kinase inhibitor. Lung Cancer (2018) 126:112–8. doi: 10.1016/j.lungcan.2018.10.023 30527174

[45] Arrieta O Barron F Maldonado F Cabrera L Corona-Cruz JF Blake M . Radical consolidative treatment provides a clinical benefit and long-term survival in patients with synchronous oligometastatic non-small cell lung cancer: A phase ii study. Lung Cancer (2019) 130:67–75. doi: 10.1016/j.lungcan.2019.02.006 30885354

[46] De Ruysscher D Wanders R Hendriks LE van Baardwijk A Reymen B Houben R . Progression-free survival and overall survival beyond 5 years of nsclc patients with synchronous oligometastases treated in a prospective phase ii trial (Nct 01282450). J Thorac Oncol (2018) 13(12):1958–61. doi: 10.1016/j.jtho.2018.07.098 30253974

[47] Levy A Hendriks LEL Berghmans T Faivre-Finn C GiajLevra M GiajLevra N . Eortc lung cancer group survey on the definition of nsclc synchronous oligometastatic disease. Eur J Cancer (2019) 122:109–14. doi: 10.1016/j.ejca.2019.09.012 31671363

[48] Schouten LJ Rutten J Huveneers HA Twijnstra A . Incidence of brain metastases in a cohort of patients with carcinoma of the breast, colon, kidney, and lung and melanoma. Cancer (2002) 94(10):2698–705. doi: 10.1002/cncr.10541 12173339

[49] Shin DY Na II Kim CH Park S Baek H Yang SH . Egfr mutation and brain metastasis in pulmonary adenocarcinomas. J Thorac Oncol (2014) 9(2):195–9. doi: 10.1097/JTO.0000000000000069 24419416

[50] Zhang I Zaorsky NG Palmer JD Mehra R Lu B . Targeting brain metastases in alk-rearranged non-Small-Cell lung cancer. Lancet Oncol (2015) 16(13):e510–21. doi: 10.1016/S1470-2045(15)00013-3 26433824

[51] Mulvenna PM Holt T Stephens R . Response to “Diagnosis-specific prognostic factors, indexes, and treatment outcomes for patients with newly diagnosed brain metastases: A multi-institutional analysis of 4,259 patients.” (Int J radiat oncol biol phys 2010:77:655-661). Int J Radiat Oncol Biol Phys (2011) 81(4):1194. doi: 10.1016/j.ijrobp.2010.09.045 21106307

[52] Magnuson WJ Lester-Coll NH Wu AJ Yang TJ Lockney NA Gerber NK . Management of brain metastases in tyrosine kinase inhibitor-naive epidermal growth factor receptor-mutant non-Small-Cell lung cancer: A retrospective multi-institutional analysis. J Clin Oncol (2017) 35(10):1070–7. doi: 10.1200/JCO.2016.69.7144 28113019

[53] Bauml JM Mick R Ciunci C Aggarwal C Davis C Evans T . Pembrolizumab after completion of locally ablative therapy for oligometastatic non-small cell lung cancer: A phase 2 trial. JAMA Oncol (2019) 5(9):1283–90. doi: 10.1001/jamaoncol.2019.1449 PMC6624820 31294762

[54] Dovedi SJ Adlard AL Lipowska-Bhalla G McKenna C Jones S Cheadle EJ . Acquired resistance to fractionated radiotherapy can be overcome by concurrent pd-L1 blockade. Cancer Res (2014) 74(19):5458–68. doi: 10.1158/0008-5472.CAN-14-1258 25274032

[55] Theelen W Peulen HMU Lalezari F van der Noort V de Vries JF Aerts J . Effect of pembrolizumab after stereotactic body radiotherapy vs pembrolizumab alone on tumor response in patients with advanced non-small cell lung cancer: Results of the pembro-rt phase 2 randomized clinical trial. JAMA Oncol (2019) 5(9):1276–82. doi: 10.1001/jamaoncol.2019.1478 PMC6624814 31294749

[56] Jang RW Caraiscos VB Swami N Banerjee S Mak E Kaya E . Simple prognostic model for patients with advanced cancer based on performance status. J Oncol Pract (2014) 10(5):e335–41. doi: 10.1200/JOP.2014.001457 25118208

[57] Sperduto PW Yang TJ Beal K Pan H Brown PD Bangdiwala A . Estimating survival in patients with lung cancer and brain metastases: An update of the graded prognostic assessment for lung cancer using molecular markers (Lung-molgpa). JAMA Oncol (2017) 3(6):827–31. doi: 10.1001/jamaoncol.2016.3834 PMC5824323 27892978

[58] Salama JK Chmura SJ Mehta N Yenice KM Stadler WM Vokes EE . An initial report of a radiation dose-escalation trial in patients with one to five sites of metastatic disease. Clin Cancer Res (2008) 14(16):5255–9. doi: 10.1158/1078-0432.CCR-08-0358 18698045

[59] Tang X Shen Y Meng Y Hou L Zhou C Yu C . Radiation-induced lung damage in patients treated with stereotactic body radiotherapy after egfr-tkis: Is there any difference from stereotactic body radiotherapy alone? Ann Palliat Med (2021) 10(3):2832–42. doi: 10.21037/apm-20-1116 33548998

[60] Wu YL Planchard D Lu S Sun H Yamamoto N Kim DW . Pan-Asian adapted clinical practice guidelines for the management of patients with metastatic non-Small-Cell lung cancer: A csco-esmo initiative endorsed by jsmo, ksmo, mos, sso and tos. Ann Oncol (2019) 30(2):171–210. doi: 10.1093/annonc/mdy554 30596843

[61] Opitz I Patella M Payrard L Perentes JY Inderbitzi R Gelpke H . Prognostic factors of oligometastatic non-Small-Cell lung cancer following radical therapy: A multicentre analysis. Eur J Cardiothorac Surg (2020) 57(6):1166–72. doi: 10.1093/ejcts/ezz384 32011665

[62] Mitchell KG Farooqi A Ludmir EB Corsini EM Sepesi B Gomez DR . Pulmonary resection is associated with long-term survival and should remain a therapeutic option in oligometastatic lung cancer. J Thorac Cardiovasc Surg (2021) 161(4):1497–504 e2. doi: 10.1016/j.jtcvs.2020.02.134 32331820

[63] Ambrogi MC Lucchi M Dini P Melfi F Fontanini G Faviana P . Percutaneous radiofrequency ablation of lung tumours: Results in the mid-term. Eur J Cardiothorac Surg (2006) 30(1):177–83. doi: 10.1016/j.ejcts.2006.03.067 16723242

[64] Dupuy DE Zagoria RJ Akerley W Mayo-Smith WW Kavanagh PV Safran H . Percutaneous radiofrequency ablation of malignancies in the lung. AJR Am J Roentgenol (2000) 174(1):57–9. doi: 10.2214/ajr.174.1.1740057 10628454

